# The Improvisational State of Mind: A Multidisciplinary Study of an Improvisatory Approach to Classical Music Repertoire Performance

**DOI:** 10.3389/fpsyg.2018.01341

**Published:** 2018-09-25

**Authors:** David Dolan, Henrik J. Jensen, Pedro A. M. Mediano, Miguel Molina-Solana, Hardik Rajpal, Fernando Rosas, John A. Sloboda

**Affiliations:** ^1^Guildhall School of Music and Drama, London, United Kingdom; ^2^Department of Mathematics, Centre of Complexity Science, Imperial College London, London, United Kingdom; ^3^Institute of Innovative Research, Tokyo Institute of Technology, Yokohama, Japan; ^4^Department of Computing, Imperial College London, London, United Kingdom; ^5^Data Science Institute, Imperial College London, London, United Kingdom; ^6^Department of Electrical and Electronic Engineering, Imperial College London, London, United Kingdom

**Keywords:** improvisation, classical performance, musical communication, neural complexity, motion analysis, state of mind, classical improvisation, flow

## Abstract

The recent re-introduction of improvisation as a professional practice within classical music, however cautious and still rare, allows direct and detailed contemporary comparison between improvised and “standard” approaches to performances of the same composition, comparisons which hitherto could only be inferred from impressionistic historical accounts. This study takes an interdisciplinary multi-method approach to discovering the contrasting nature and effects of prepared and improvised approaches during live chamber-music concert performances of a movement from Franz Schubert's “Shepherd on the Rock,” given by a professional trio consisting of voice, flute, and piano, in the presence of an invited audience of 22 adults with varying levels of musical experience and training. The improvised performances were found to differ systematically from prepared performances in their timing, dynamic, and timbral features as well as in the degree of risk-taking and “mind reading” between performers, which included moments of spontaneously exchanging extemporized notes. Post-performance critical reflection by the performers characterized distinct mental states underlying the two modes of performance. The amount of overall body movements was reduced in the improvised performances, which showed less unco-ordinated movements between performers when compared to the prepared performance. Audience members, who were told only that the two performances would be different, but not how, rated the improvised version as more emotionally compelling and musically convincing than the prepared version. The size of this effect was not affected by whether or not the audience could see the performers, or by levels of musical training. EEG measurements from 19 scalp locations showed higher levels of Lempel-Ziv complexity (associated with awareness and alertness) in the improvised version in both performers and audience. Results are discussed in terms of their potential support for an “improvisatory state of mind” which may have aspects of flow (as characterized by Csikszentmihalyi, 1997) and primary states (as characterized by the Entropic Brain Hypothesis of Carhart-Harris et al., 2014). In a group setting, such as a live concert, our evidence suggests that this state of mind is communicable between performers and audience thus contributing to a heightened quality of shared experience.

## Introduction

### Motivation

Although classical music performance is recognized as a creative practice, its parameters have been restricted by a longstanding ethos of “faithfulness to the composer's score” which limit the bounds of acceptable deviation (Leech-Wilkinson, 2016). This ethos has dominated classical music performance since the late nineteenth century. However, historical research has revealed that Western art-music composers from Bach, through Mozart and Beethoven and onwards into the romantic era expected and encouraged performers to creatively depart from the score in a far more radical way than is common today, including the insertion of new notes (Eigeldinger, 1986; Hamilton, 2008).

In these earlier times improvisation was not only encouraged, but it was believed by many to be an essential component of complete musicianship and mastery. For instance, improviser and composer Johann Nepomuk Hummel (1778–1837) recommended “free improvisation in general and every respectable form to all those for whom [music] is not merely a matter of entertainment and practical ability, but rather principally one of inspiration and meaning in their art” (quoted in Goertzen, 1996, p. 305)^1^. Hummel stated in 1828 that this matter was urgent, and cautioned, “Even if a person plays with inspiration but also from a written score, he or she will be much less nourished, broadened, and educated than through the frequent immersion in free fantasy practiced in the full awareness of certain guidelines and directions, even if this improvisation is only moderately successful” (Goertzen, 1996).

In recent years there has been a renaissance and awakening of interest in practicing, teaching, learning, and researching Western classical music improvisation (e.g., Berkowitz, 2010). For example, while in most high profile international competitions improvising repeats, preludes, fermata points or cadenzas is still considered by competitors to be an unwise risk, the Bach international piano competition in Leipzig (under the artistic direction of Robert Levin) encourages it explicitly, by saying in the instructions to competitors that extemporized repeats are welcome and encouraged (http://www.bachwettbewerbleipzig.de/en/bach-competition/competition-programme-2018).

Improvisation is beginning to find its way into the pedagogical curriculum for music (Azzara and Snell, 2016). However, this is still sufficiently uncommon for Shehan Campbell et al. (2014) to be able to conclude “That the majority of music students graduate with little to no experience, let alone significant grounding, in the essential creative processes of improvisation and composition represents one of the most startling shortcomings in all of arts education”.

The re-insertion of this “improvisatory approach” into classical music professional practice is sufficiently new that the contemporary practitioners of this approach have predominantly been schooled in the mainstream approach of score faithfulness, and switch between the two approaches in their artistry, thus affording researchers the possibility of comparing the nature and effects of improvised performances with “conventional” performances of the same pieces by the same performers.

It is this unique juncture in artistic history which has motivated and enabled us to investigate exactly what it is that differentiates the improvisatory approach to performance from the conventionally prepared one, in its nature, its cognitive and neural underpinnings, and its effects.

### Background

Most of the recent scientific investigations into musical improvisation have centered on jazz. These studies have analyzed improvisation using tools from neuroscience (Donnay et al., 2014; Pinho et al., 2014; Lopata et al., 2017), musicology (Norgaard, 2011, 2014) and psychology (Tervaniemi et al., 2016; Love, 2017) Although these research efforts are relevant to broaden our understanding of improvisation in music, it is not straightforward how to isolate the effect of improvisation as there is no natural baseline to compare with. Improvisation is a fundamental and omnipresent ingredient in Jazz music and therefore is to be expected that Jazz musicians and listeners will have a preference for it. In contrast, in classical music the default choice for the last 100 years is to perform without improvisational elements.

The distinctive feature of classical music improvisation (at least in the present day) is the existence of a strong canonical form (usually represented by a written score and well known within the community of listeners) from which improvisation is a deliberate deviation. Faithfulness to the canonical score is also a valid artistic response, whereas within other artistic forms, such as Jazz, the faithful rendition of a “cover” melody would be considered of little artistic interest. For a more detailed discussion of the nature of classical improvisation see Dolan et al. (2013, pp. 1–6). Although very few existing studies examine improvisation in the context of Western classical art-music, a notable exception is Després et al. (2017), who explore strategies applied by five internationally recognized classical music solo improvisers by means of analyzing semi-structured retrospective interviews. However, this study did not gather any data from actual performances and therefore sheds only indirect light on performance characteristics and audience response.

Improvisation is a listener-directed art, and so it is critical to our understanding of it to know what effect it has on listeners/audiences. There are many anecdotal and historical accounts of the power and impact of improvisatory performances of classical music, such as the report of the “tumultuous applause” that greeted a 30 min improvisation by Mozart in Prague in 1787 (Johann Nepomuk Stiepanek, reported in Abert 2007, p. 827). However, very few studies have attempted to investigate the impact on traditional concert audiences of listening to live performances of classical music that vary in their expressive intent. Some studies have measured the subjective responses of audience members via questionnaires and/or interviews (Pitts, 2005; Thompson, 2006, 2007; Pitts and Spencer, 2007; Dobson, 2008), but none of them directly addresses responses to the improvised or spontaneous elements of the performance. Also, the substantial neuroscience literature on the relationship between music and language (see Hutka et al., 2013 and references therein) focuses on sensory and semantic processing of individuals, and does not address the interaction between performers and listeners. Studies measuring brain activity of individuals listening to improvised Western classical music hardly exist. The only available data to date come from a pilot study reported by Dolan et al. (2013) (further analyzed in Wan et al., 2014), which studied the effect of conventional and improvised live performances of pieces from the classical repertoire on both musicians and listeners. The results showed significant differences in performance features, subjective experience and brain activity between prepared and improvised performance, providing initial evidence that improvised performances of the classical repertoire can heighten musical effectiveness and audience response.

### Understanding the improvisatory approach as a state of mind

In this study we explore and elaborate the notion that improvisational activity induces a particular state of mind in performers and audience different from that habitually present in prepared performances. By “state of mind” we refer to a distinct mental and neural configuration which may be maintained for a period of time, and which involves specific cognitive and affective components. We seek to shed light on how might such a state be best characterized, how it relates to other states of mind, and how and in what ways such a state is communicable or transferable to listeners. We consider two separate but related lines of prior empirical enquiry as of particular relevance.

One is the body of investigation into the multidimensional phenomenon known as *Flow*, as introduced into Psychology by Csikszentmihalyi (1975). Originally described as “the holistic sensation that people feel when they act with total involvement,” this state of mind is characterized by full engagement, sensation of creativity combined with enhanced well-being, effortless control and concentration, a sense of having clear goals and full presence in one's performance together with a reduced awareness of the time passing (Chirico et al., 2015). Moreover, *flow* is to be distinguished from creativity, the latter meaning the creation of novelty while the former refers to an effortless yet highly focused state of consciousness (Csikszentmihalyi, 1996).

There exists a close relationship between the state of flow and music experience. In fact, it has been claimed that music is the activity in which it is easiest to reach an experience of flow (Csikszentmihalyi, 1997; Lowis, 2002). Chirico et al. (2015) review recent investigations into the relationship between music and flow, covering musical performance, composition and listening. Improvisation as a source of flow has been neglected, although Després et al. (2017), suggest that Berkowitz (2010) characterization of a “witness” state of mind in the solo classical improvisation of Robert Levin and Malcolm Bilson may hint at elements of flow. In the “creator” state, a musician develops the improvisation consciously and deliberately, using declarative knowledge. In the “witness” state, the improviser is more akin to a spectator of his or her own unfolding improvisation which emerges through implicit procedural knowledge. However, in both Berkowitz (2010) and Després et al. (2017) investigations of solo classical improvisation, the data came from extended retrospective interviews separated from any specific performance, and thus not optimal for uncovering evidence of flow states which, by definition, are “in the moment.” In addition there was no consideration in any prior studies of how such states may be shared between musicians in group improvisation or communicated to listeners. There is thus much still to discover about the way that different levels of conscious awareness guide the real-time decision making process.

A second line of enquiry comes from work into the “entropic brain hypothesis” (Carhart-Harris et al., 2014). Combining recent neuroimaging findings with psychoanalytic concepts, the EBH distinguishes between two different styles of human cognition: *secondary states* that are characteristic of the experience of contemporary adult humans, and *primary states* to which the mind regresses under specific conditions, e.g., in response to severe stress, psychedelic drugs or in REM sleep. Physiologically, primary states are characterized by an elevated entropy in various brain function that is manifested in e.g., fMRI or EEG measurements with high signal complexity, which correlates with diversity and richness of experiential content. Conversely, entropy is suppressed in secondary states generating measurements with lower signal complexity and hence more regular and stable cognitive processes, hence enabling metacognitive functions including reality-testing and self-awareness.

The EBH further hypothesize that primary states are evolutionarily older than secondary states:

“*… the mind has evolved (via secondary consciousness upheld by the ego) to process the environment as precisely as possible by finessing its representations of the world so that surprise and uncertainty (i.e., entropy) are minimized. … In contrast, in primary states, cognition is less meticulous in its sampling of the external world and is instead easily biased by emotion, e.g., wishes and anxieties*.” (Carhart-Harris et al., 2014)

However, although primary consciousness may be a sub-optimal mode of cognition, it seems to be more than a mere psychological atavism. Plenty of reports show how events involving primary states can bring deep experiences and have profound therapeutic effects (Griffiths et al., 2008; Carhart-Harris and Nutt, 2010; MacLean et al., 2011). In effect, the high entropy of primary states seems to allow overcoming the inability to think and behave in a flexible manner, narrow-mindedness and aggressive self-critical attitudes.

Although the EBH was developed to provide a theoretical basis for therapeutic uses of psychedelic drugs, it is natural to ask if it is applicable to the domain of musical experience, and in particular musical improvisation. Is the improvisational state of mind a primary state? Could one find traces of primary states in musicians and audience during improvisational activities?

### Scope of the present study

The present study aimed to answer these questions, building on Dolan et al. (2013), and addressing a number of key shortcomings and limitations.

A first limitation of Dolan et al. (2013) study was that it employed a traditional EEG analysis to track the activation of various cortical areas related to alpha and beta frequency bands. In contrast, the present study focuses on the *Lempel-Ziv complexity* (LZ) of the EEG signals, which is the preferred method for studying brain entropy and signal complexity within the EBH framework (Carhart-Harris, 2018). The method was introduced by Abraham Lempel and Jacob Ziv to study the complexity of binary sequences (Ziv, 1978), and was later extended for EEG signals to study epilepsy (Radhakrishnan and Gangadhar, 1998) and depth of anesthesia (Zhang et al., 2001). When characterizing states of mind, LZ is higher in subjects during wakeful rest than in subjects during sleep or general anaesthesia (Casali et al., 2013; Schartner et al., 2015). LZ is also higher than normal when the brain is under the effect of psychedelic substances (Schartner et al., 2017). Even at the individual level, LZ is correlated with a more vivid imagination and ego dissolution (Schartner et al., 2017). Also, the brain's response to a given stimulus scores higher LZ when the stimulus is more meaningful to the viewer (Boly et al., 2015). In summary, there is strong evidence in the literature that suggests that LZ is a reliable indicator of awareness and alertness.

A second limitation of the pilot study concerned the composition of the audience, which was primarily drawn from highly-trained students and staff of a conservatoire. It is possible that the significant effects of improvisation could result from a sophisticated level of musical training and awareness, and would not be generalizable to a broader public. In order to better characterize the impact of improvisation on the listening population, an audience containing a wider range of musical knowledge and experience is needed.

Thirdly, informal observations by Dolan et al. (2013) suggested that musicians engaged in larger bodily gestures during the improvised performance than during conventional performances. It is possible that some of the audience effects observed were not due to the differences in sound parameters as such, but the visual aspects of the performance. To explicitly assess the differential effects of sound and vision on audience response, formal measurement of performer movement would be needed, as well as comparing responses of audience members of those who could hear but not see the performances, with those who could both see and hear.

Fourthly, the pilot study examined performance data from only two composers, the baroque composer Telemann, and the post-romantic/impressionist composer Ravel. Analysis of the performance related parameters revealed that although the performers performed both works with style and period-specific approach to tone and articulations (in both performance modes), they used similar performing strategies when applying improvisational approach to performing both Telemann and Ravel's works. During the improvised performances more attention was given to longer-term gestures, phrasing was more coherent structurally while at the same time inserting spontaneous but shared extemporized passages. This might be seen as an unexpected result, since improvisation, because of its unplanned nature, is often presumed to be unstructured and less coherent than non-improvised performance. The generality of these characteristics would be better established by investigating their occurrence in other classical styles, such as the early romantic period typified by a composer such as Franz Schubert.

Fifthly, while gathering verbal feedback from the audience, and brain measurements from both performers and audiences, the Dolan et al. (2013) study did not formally capture the insights and impressions of the performers themselves. For a fuller understanding of the parameters of an improvisatory state of mind, objective measures (of performance parameters and brain activities) should be compared with the subjective experience of the players.

### Research questions and paper structure

The primary questions which motivates the current study are

1. Does the improvisational act induce a different state of mind in performers, and is it transferable to listeners? Following from that,
2. such state exists, how can we best characterize it?

At a more detailed level, further elaborating question 2 in respect of the key concepts of flow and the EBH:

3. Do performers' subjective accounts of their improvisatory experiences contain elements indicative of a flow experience?
4. Are there quantitative signatures of a shift from a secondary toward a primary state of cognition when comparing the brain activity during the prepared and improvised performances? In particular, can one find significant differences in terms of the LZ complexity of the EEG signals of musicians and audience?

Finally, as control questions aimed at resolving the limitations of earlier work discussed in section Scope of the Present Study above:

5. Dothe body movements of musicians as visually experienced by audience members affect the magnitude of their response to the improvised performances? This will help clarify which medium is the basis of the communication of the contrasting states.
6. Does the level of musical training or knowledge of audience members affect their response to the improvised performances? Can the effects only be manifested between trained people?
7. Do the objective performance characteristics that distinguish improvised performances of Telemann and Ravel extend to the music of a different period exemplified by Schubert? In particular, is there evidence of a greater degree of coherence and longer-term phrasing in the improvised version of Schubert?

Posing these questions has led us to the use of a combination of different methodologies in an interdisciplinary approach to capturing and analyzing multiple aspects of concert performances of items from the classical chamber ensemble repertoire. The design of the study allows us to measure the following features of conventional and improvised performances (given here in the order in which they are treated in the results section):

- (A) sonic and performance related parameters characteristics of the performances (notes played, and the timing, dynamics, and timbral qualities of those notes).
- (B) Post-performance assessments by the musicians themselves;
- (C) Continuous body motion tracking;
- (D) Post-performance audience ratings;
- (E) Real-time continuous monitoring of brain activity (EEG) of performers and audience members;

This order or presentation represents a progression from examining aspects of the performances and the performer experience, to examining the audience experience, and finally the co-ordination between performers and audience.

## Methods

### Participants

Musical performers consisted of a professional trio—Kate Smith (voice), Rosie Bowker (flute) and Thibault Charrin (piano)—expert in classical improvisation, recruited and mentored by the 1st author. In particular their improvisatory practice was deeply informed by the performance practice developed over a lengthy period in the context of an advanced pedagogical center headed by the 1st author. The performers, although now independent professional practitioners had experienced extensive tuition and professional development in that context.

The invited audience comprised 22 adults, mainly postgraduate students and staff from the two UK academic institutions involved in the study. They contained individuals with a wide range of experience with, and training in, classical music. This was ensured by asking potential audience members to complete a pre-screening questionnaire, with questions about musical experience (for details, see results of questionnaire data).

Informed consent was obtained through a letter of invitation to all participants outlining what would take place in the experiment and asking them to confirm their acceptance of the invitation. Once accepted, a small subset of the audience were invited in writing to participate in the EEG study. Of the initial four audience members invited, one declined, and was replaced by a fifth who accepted. Performers gave explicit written permission for their identity to be revealed.

### General procedure

The experiment took the form of a live chamber music concert on 21 March 2017. It took place in the Data Observatory at the Data Science Institute, Imperial College London (institution of the 4th author) with the aim of using its motion capture facilities, in the presence of an invited audience, all of whom had agreed in advance to be participants in the experiment. A Yamaha C-7 grand piano was hired to ensure the closest approximation to a fully professional concert. The seating was arranged such that half the audience could only hear but not see the performers. The size of the audience was the maximum feasible given the available space in the laboratory, in addition to the performers, the research team, and the scientific and musical equipment in place.

During the experiment each piece was performed twice: once in what the performers themselves chose to describe through their shared professional understanding as a “strict” mode (corresponding to a prepared interpretation), and once in what they described as a “let-go” mode (corresponding to the improvisatory approach in which they had been mentored). In the strict / prepared mode the players focused mainly on controlling technical precision, timing co-ordination, accuracy of the score's details, avoiding risks, while at the same time creating the most convincing and expressive performance possible. In contrast, during the let-go / improvised performance the players were asked to play freely, as they would do for friends, expressing themselves spontaneously and not putting an imperative focus on “no wrong notes.” Note that the let-go performance still requires thorough knowledge of the written work, its harmonic and stylistic language and at the same time the ability to deviate from the written text in an unplanned coordination with the other ensemble partners. Moreover, the musicians were not operating according to any explicitly articulated set of rules for guiding these improvised deviations from the score. The order of the prepared and improvised performances was randomly varied from item to item, and this order was known only to the performers, who decided the order on the spur of the moment (i.e., audience members were unaware of which version was played each time).

The audience was briefed by one of the researchers that they were about to hear a sequence of pairs of trio performances that would involve some elements of improvisation. All members of the audience were asked to provide verbal responses via a questionnaire which was distributed prior to the start of the performance. After each performance members of the audience were given a short time to rate it for the degree to which they detected or experienced five qualities: improvisatory in character, innovative in approach, emotionally engaging, musically convincing, and risk-taking. These questions were identical to the ones used in the Dolan et al. (2013) study, Responses were made using a six-point Likert scale, ranging from “not at all/none” to “totally/completely.”

The continuous movements (3-dimensional positions of up to 20 joints) of the three performers were captured by means of an existing motion tracking system formed by five Microsoft Kinects devices distributed circularly around the performers (further technical specifications are given in the results section Continuous Body Motion Tracking below).

EEG brain activity of four audience members as well as the three performers were captured with seven high-performance EEG recorders using 19 electrodes for each person (further technical specifications are given in the results section dedicated to EEG data analysis below). Two of these audience members could both see and hear the performances, the other two could hear but not see them. Within each pair, one participant had a high degree of training in classical music, the other a low degree.

High quality audio and video recordings of the performances were made by means of two HD videocameras located in different positions.

## Data analysis and results

In this paper we confine our analysis to data from two performances of the opening Andantino section of Franz Schubert's “*Der Hirt Auf Dem Felsen” (The Shepherd on the Rock)* Op 129. Within the pieces measured during our experiment, drawn from the existing repertoire of the performers, this was the piece which the performers judged best realized their differential intentions for the two performance modes, and provided sufficient data for an intensive analysis.

The analysis proceeds from sonic and musical features of the performance, as experienced and characterized by the musicians involved, through the visual features of those performances captured by movement, leading to the explicit audience response to these performances, and concluding with the neurophysiological data examining relationships across and between performers and audience at a level beneath the conscious and explicit.

### Sonic and performance related parameters characteristics of the performances

This section presents an analysis of the performance-related parameters of the prepared and the improvised versions. This analysis was undertaken by the 1st author with the aid of repeated critical listening (jointly with the performers) and *Sonic Visualizer software*^2^ which provided a visual trace of key physical characteristics of the performances.

Below we first summarize some overall characteristics of the performances, and then present a more detailed analysis of three particular—yet characteristic—moments where the musicians spontaneously took enhanced risks in the improvised version—by deviating from the score's instructions in terms of timing, dynamics, and timbre, actual extemporized notes, or a combination of all three. The audio/video clips of each moment in the two performances are added as Supplementary Files (Videos 1–6) respectively where the first file of each pair is extracted from the prepared performance and second is the improvised).

In what follows in this analysis, objective measures (duration, intensity, frequency) are interpreted in the light of inter-subjective judgment of the first author and performing musicians. Thus, all evaluative remarks (terms such as “better”) reflect the joint musical judgement of the individuals concerned.

#### General observations

When comparing the prepared and improvised performances we found significant differences in six features, the first four of which pertain to physically measurable sonic and temporal characteristics of the performances, and the last two of which pertain to structural features of the performance.

##### Timbre

In the improvised version there is a wider range of timbre changes both individually and in the group orchestration (see Example 1 below).

##### Speed (tempo/duration)

The improvised performance is objectively slower (average crotchet/quarter note = 88 bpm) than the prepared one (average crotchet/quarter note = 92 bpm). However, the critical listening confirmed that despite the slower tempo—in absolute terms—of the improvised version, it gave the subjective impression of being faster and more “forward going.”

##### Dynamics

In the improvised version the dynamic diversity is larger compared with the prepared version. For example, the intensity in the prepared version of the start of the performance (bars 7–9) varies between −17.75 and −14.40 dB, whereas in the improvised version it varies between −31.14 and −14.77 dB (a range 14 dB bigger) (see Examples 1 and 2 below).

##### Pulse, meter and metrical division

The improvised performance contains more longer-term phrasing gestures. These are better coordinated between performers and more in line with Schubert's written instructions (dynamics, timing and expression) compared with the prepared mode. For instance, there is one phrasing slur mark in Schubert's score, running from the last beat of bar 19 to the first beat of bar 21. Expert critical listening, supported by the Sonic Visualizer data, confirms that the improvised performance follows this instruction more closely than the prepared version. In the improvised version there are smoother timing and dynamic transitions from bar 19 to 20, and 20 to 21, whereas in the prepared version there are discontinuities which emphasize individual crochet beats and the start and end of each bar unit, thus breaking the indicated phrasing.

Moreover, whole-bar beats and hyper-measures^3^ of two bars are clearly heard (and seen) in the improvised version, while hardly existing in the prepared version. This might account for the impression of a more forward going musical movement, felt during the critical listening sessions, while the prepared version is at times more fragmented. An example of this is discussed in more detail below (Example 2).

##### Risk taking

In the prepared version, the musicians perform the written instructions literally, and thus make it more predictable (easier to anticipate what happens next). In the improvised version they spontaneously deviate from the text by means of timing, extended dynamics and timbre as well as extemporized notes, making the performance less “safe” to manage. And yet, repeated critical listening concluded that it is in the latter version where the performers were better coordinated in key moments (end of phrases, moments of harmonic resolution and significant harmonic changes).

##### “Mind-reading” during shared extemporized gestures

By “mind reading” we mean moments where one musician deviates from the score by extemporizing notes and another extemporizes in response instantly, creating together an unplanned, yet coherent joint musical gesture that reaches a final goal point together. Such moments only occur during the improvised performance (compare Videos 5 and 6), suggesting heightened listening. This is also confirmed on in the musicians' reports.

#### Detailed analysis of specific representative examples

Below we present an analysis of three indicative examples, illustrating in more detail the artistic differences between the prepared and improvised performances. There are more similar examples throughout the performance which space precludes mentioning, but which will form the basis of a more detailed musicologically oriented publication (in preparation).

##### Example 1

**Figure 1:**
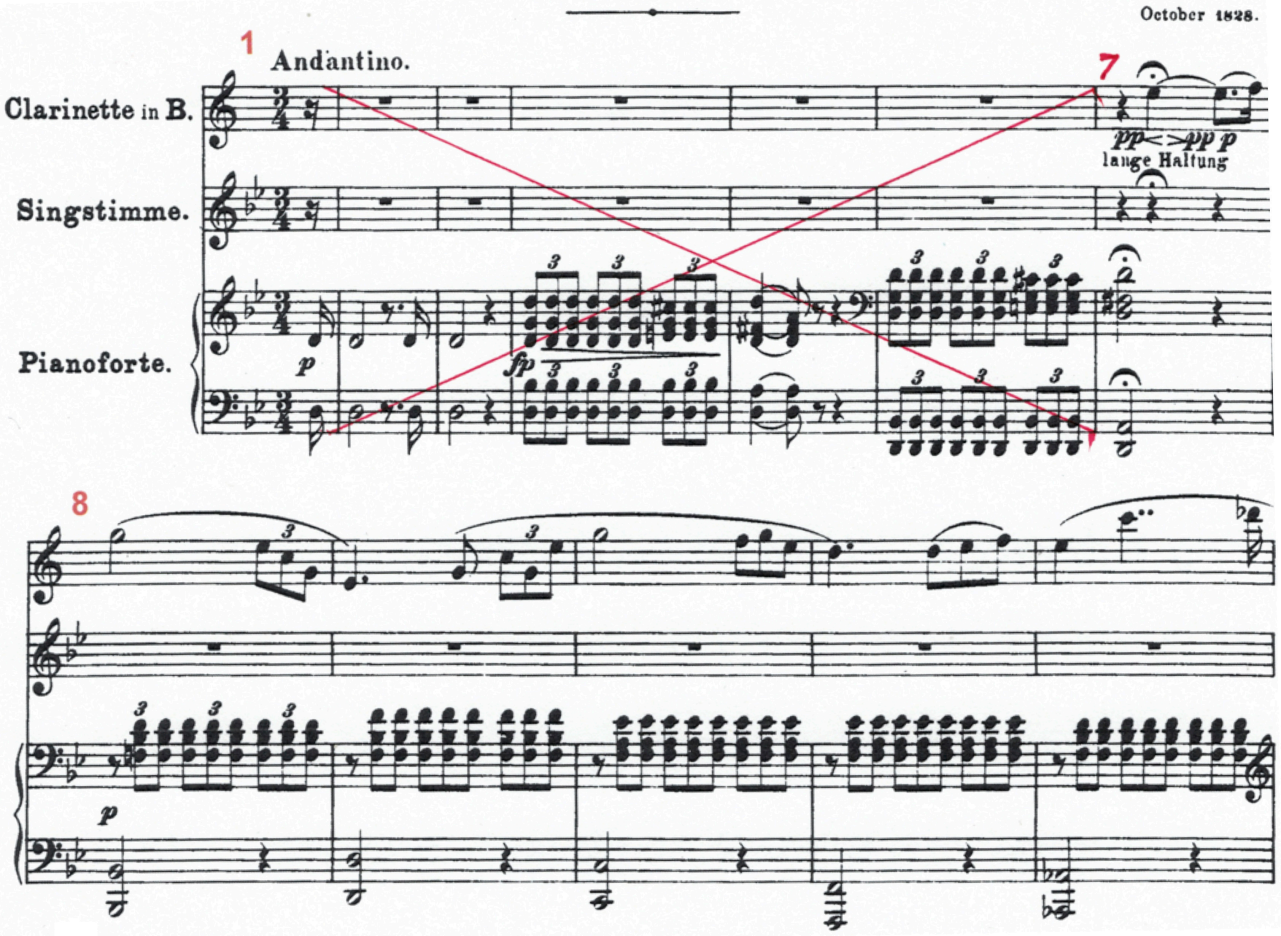
Score of Schubert's Shepherd on the Rocks, Op 129^5^, Bars 7–8. The very opening of the performance. [Associated video/audio clips are 1 [prepared bar 7 - bar 8 beat 1], 2 [improvised bar 7 - bar 8 beat1], 3 [prepared bars 8–12] 4 [improvised bars 8–12].

This tiny 3-note long flute solo playing (see the corresponding score in Figure 1, bars 7-8) is a microcosm of the improvisational approach which permeates the entire performance and illustrates to a greater or lesser extent all 6 features outlined in the “general observations section.” We will take them one by one and show how they are manifested in this segment.

**Figure 2:**
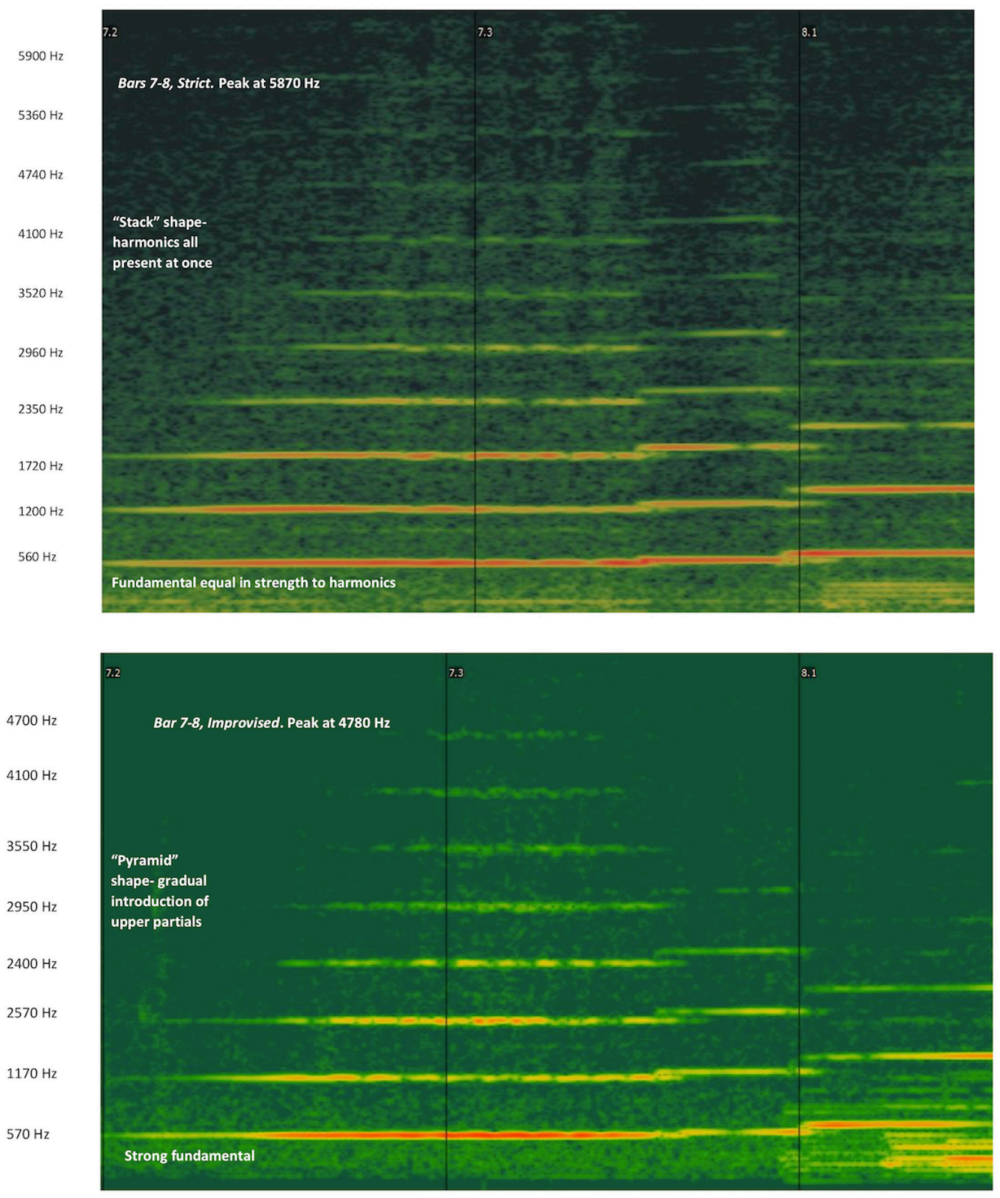
Flute harmonics up to 5,400 Hz (fundamental frequency at the bottom plus the first 10 overtones above). Prepared performance in the top figure, improvised performance in the bottom one. 7.2 and 7.3 indicate the 2nd and 3rd beats of bar 7 respectively.

*Timbre*. We chose this point for illustrating the timbral element because it is the only moment where timbre is clearly analysable by the sonic visualizer software, as only one instrument is playing. The two spectrographs in Figure 2 visually illustrate timbral characteristics to be heard in the audio clips of the two performances. In the improvised performance there is a gradual evolution of the timbre during the first note played (reflected in the harmonics appearing gradually), while in the prepared version the first three harmonics appear more strongly together from the outset. In the improvised version the fundamental frequency as well as the lower harmonics are stronger (manifested by the thicker and more emphasized colors of these first four spectrograph lines in the improvised version, as seen in both spectrographs of Figure 2). The higher harmonics are relatively less present in the improvised version, comparing with the prepared version. (Peak of the harmonics in the prepared version is 5,380 Hz, in the improvised one (4,780 Hz). This contributes to the improvised version having a softer timbre (less emphasized higher harmonics) on the flute's *e flat* and *f*.

The tone quality in the prepared version is as excellent as it is in the improvised version (with hardly any use of vibrato) stable and in full control, suggesting a choice rather than a “better” performance.

*Tempo/duration*. There is no clear tempo at the beginning of the improvised version, which creates an “out of time” effect in the solo flute's entry. It is achieved by the significantly longer duration of the opening *d* (comparing with the prepared version) 4.15 s vs. 2.8 s respectively, fluctuations in the speed of vibrato, and a dynamic wave mentioned below.

*Dynamics*. Unlike the prepared version, in the improvised version there is an extreme dynamic range, with an unexpected additional dynamic “wave” of down and up again—this time with a narrow vibrato toward the end of the long *d*, continuing into the *e-flat* followed by the *f* without separate articulations. In the prepared version the flutist applies a milder, consistent crescendo, (without the dynamic “wave” at the end of the long *d* note).

*Pulse and meter*. Together with the fact that here is no clear beat in the opening of the improvised version, the above points mean that the gesture *e flat* ⇒ *f* is performed more as a prolongation of the *d* than a separate rhythmical event leading to a different bar. By doing so, bars 7 and 8 become one hyper-measure of two bars, with a first part (bar 7), being free, out of tempo and “out of time,” fulfilling Schubert's fermata instruction to the fullest. In the prepared version, there is a clear distinction made between bar 7 and bar 8, through the more metronomic use of accents. The distinction is further confirmed by the pianist entering in bar 8 with even quaver beats (unlike the improvised version where the pianist's meter is clearly one beat per whole bar, with one gesture every two bars). The result is a more subdivided rhythmic approach in the prepared version.

*Risk taking*. There are two risks the flutist takes within the first few seconds of the improvised performance. The first is her choice to open with a gradual evolving of the opening note's tone color mentioned above. This is a harder choice than the conventional way of approaching a tone's outset, with a higher level of risk-taking (the risk of losing the tone all together). The other risk relates to the previous point mentioned above about creating one hyper-measure of two bars (rather than relating to individual crochet beats). By so doing the flutist is taking the risk of not meeting the pianist in time for the next bar, as she “gives up” the markers of the crotchet beats to which the pianist can relate when preparing for joining the flutist in bar 8.

*Mind reading*. Despite this flutist's risky choice, they end up finding each other absolutely on time, which may suggest heightened listening and “musical mind reading” as defined above. These timing and loudness variations do not appear in the score, and are the flutist's personal spontaneous interpretation. The free rhythmical approach that the pianist takes from the start of his entry in bar 8 (as can be heard in video/audio clip 4, in contrast with 3) may be his spontaneous response to the rhythmical freedom applied by the flutist the bar just before.

**Figure 3:**
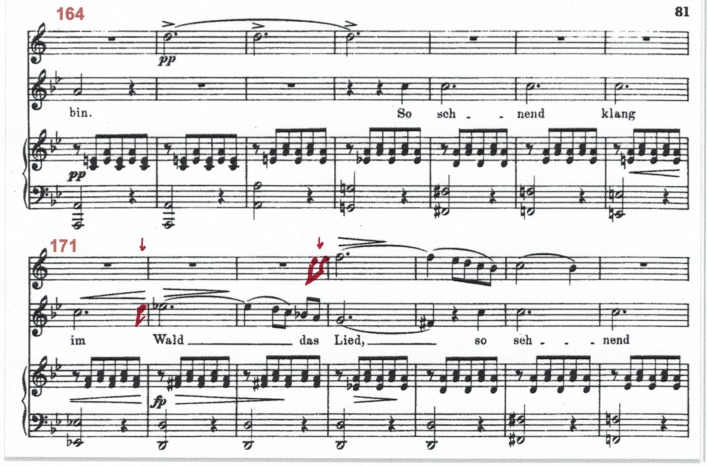
Score of Schubert's Shepherd on the Rocks, Op 129, bars 164-177. The notation added in red by the first author after the concert indicates the musicians' extemporizations in the improvised version. Related video/audio clips are 5 [prepared] and 6 [improvised].

**Figure 4:**
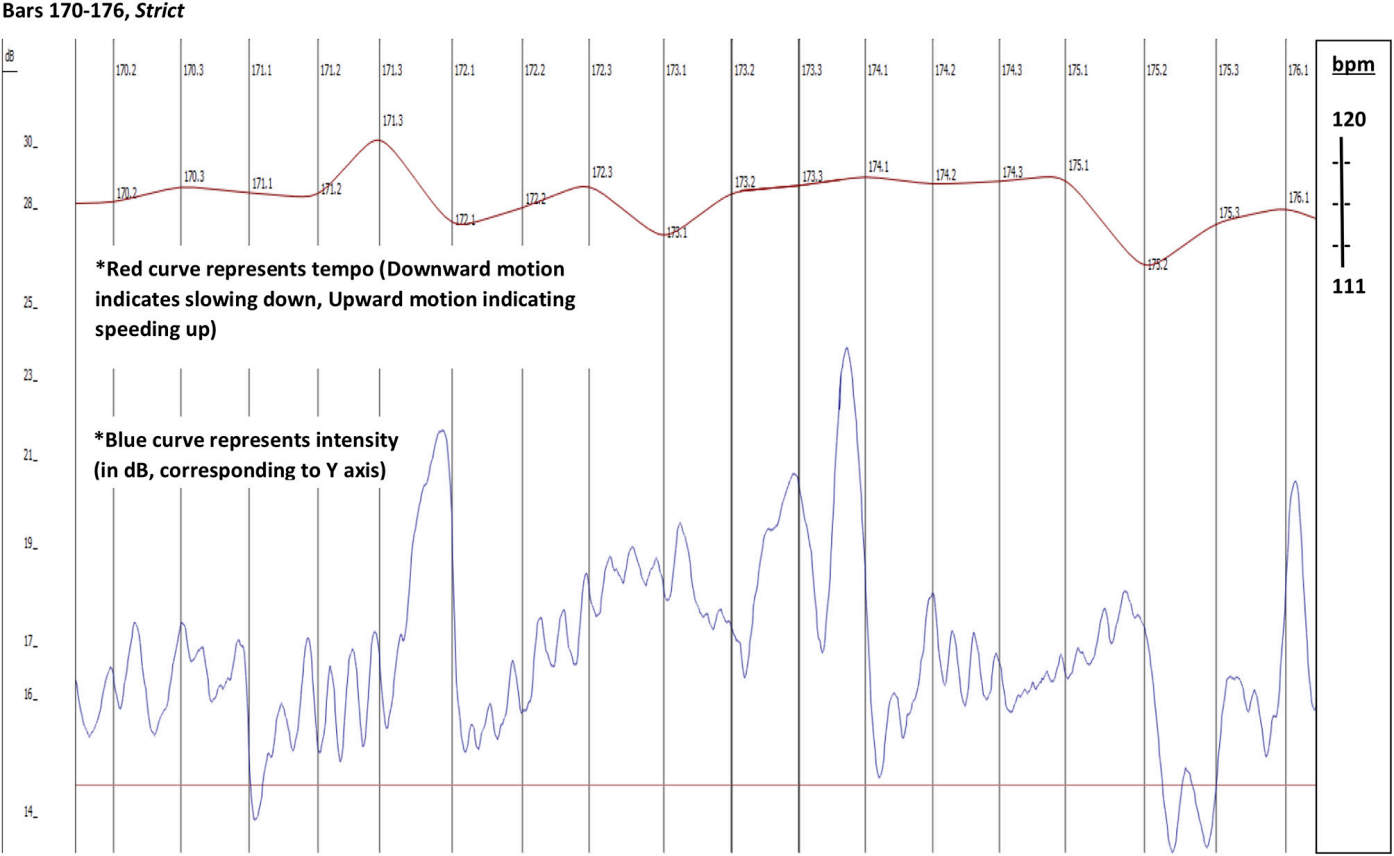
Timing (red) and dynamic (blue) profiles. Prepared performance in the upper graph, improvised performance in the bottom graph. Vertical lines indicate beat subdivisions of the 3 bars.

**Figure 5:**
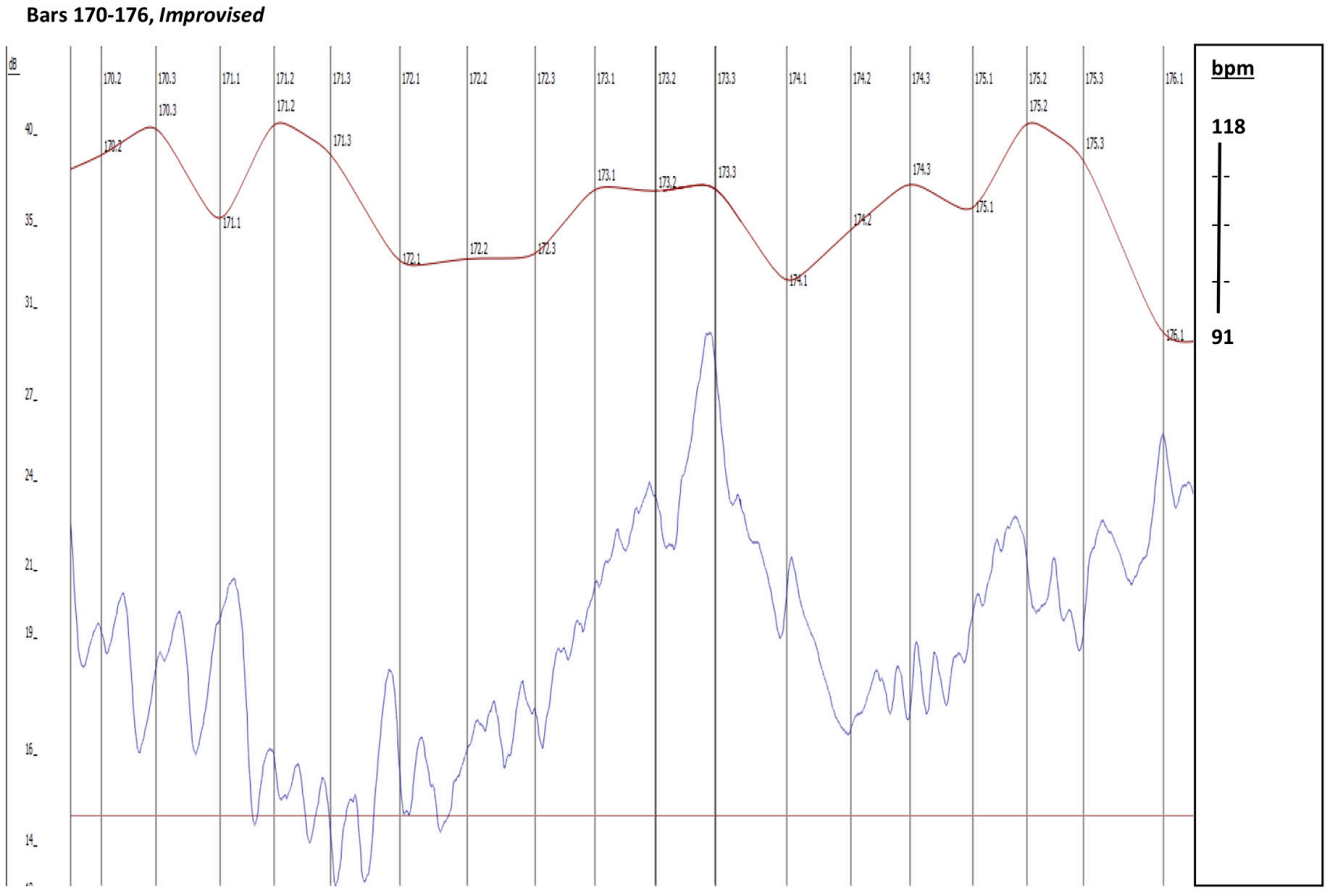
Timing and dynamic profiles bars 170–176. Timing (red) and dynamic (blue) profiles. Prepared performance in the upper graph, improvised performance in the bottom graph. Vertical lines indicate beat subdivisions of the 3 bars.

This example clearly illustrates the varieties of means of implementing an improvisational approach that do not require the extemporization of new notes, but variations in the performance parameters of composer-notated elements. This is the only example where we were able to analyse timbre in a formal way, however there are multiple examples of some of the other features. The next two examples are chosen to illustrate respectively meter and dynamics (**Figures 4**, **5**), and extemporized notes, risk-taking and mind reading (Figure 3).

##### Example 2

In this example we concentrate on tempi/durations, dynamics, pulse and meter and the inter-relations between them. We chose to look into performance related parameters in bars 8-9 (see Figure 1), as we concentrate on a specific, and early, example of a difference between prepared and improvised approach which recurs throughout the performances.

*Tempo/duration*. In the prepared version there is a greater evenness of quaver and crotchet beats comparing with the improvised rendition. The range of tempo-changes (gap between slowest and fastest) in the prepared version is slightly narrower. Also, these changes are more frequent (up-down-up-down), compared with the improvised version where there are less tempi fluctuations (just one down-up wave).

*Dynamics, pulse, meter, and phrasing—ingredients of musical flow*. In the prepared version, the frequent peaks in the loudness profile are indicative of micro-accents on each quaver beat, while in the improvised version we notice a larger and smoother wave shape, signifying the avoidance of these frequent, regular accents. This can be heard in the audio/video clips number 3 and 4 respectively and observed by the number of peaks in the curve of intensity (20 peaks in the intensity curve of the prepared version, vs. 10 in the improvised version for this segment). The overall shape of the loudness curve in the improvised performance indicates waves of dynamics in accordance with the two bars hyper-measures, resulting in a less fragmented and more flowing musical movement. The occurrence of whole-bar gestures and hyper-measures of 2-bars through large parts of the improvised performance was also identified by the musicians during the critical listening sessions, in contrast with the notion of 3 beats per bar that the musicians identified in the prepared performances (see section Post-performance Assessments below).

It is also noticeable that there is a relationship between the tempi and the loudness curves: they increase and decrease together across whole-bars units of time. Such a feature is seen as adding to the overall higher level of coherence and forward movement experienced in the improvised version, comparing with the prepared one. This is even though, according to their own reports, the musicians were much less aware of metronomic and metric (tactus) control during the improvised version, in contrast with the prepared version. Yet the actual result suggests the opposite. This dissociation between performance decisions and conscious awareness of these decisions is one of the characteristics of a state of flow (as pointed out by Després et al., 2017).

##### Example 3

This example, whose score is presented in Figure 3, is an illustration of the way in which sonic & temporal characteristics of the performances, contribute and support structural features of the performance. It also illustrates the use of extemporized notes which were not present in the original score.

The singer connects bar 171 to bar 172 with an improvised upbeat “*d*” to the following *e flat*. A bar later (173-174) the flautist extemporizes an upbeat passage to her *e flat* with all three Schubert's notes of this motive: *c* = >*d* = >*e flat*. Unlike the singer, the flautist starts her extemporized gesture *before* her entry is due in the score, and thus takes a significantly greater risk of losing her partners. Listening to bar 173 reveals the mechanism that made this possible— the pianist provides the flutist with the additional time needed to fit her extemporized responding gesture off the beat, by spontaneously slowing the tempo down, as well as playing a significant diminuendo, and thus making the *rallentando* more coherent (see the different tempo curve in Figures 4, 5 from bar 173.3 to 174.1). Following the singer's extemporized upbeat gesture at the end of bar 171, the trio maintains a noticeably slower tempo throughout bar 172.

Bars 172–175 (including) have in the base (the pianist's left hand) one minim long *d* (musically described as a “pedal”) in each of these four bars. The resulting sound effect (as exemplified in the audio clips) is of bars 172 and 173 of the improvised version being one rhythmical gesture (hyper-measure), with bars 174 and 175 being another, creating two longer gestures of two bars, where every bar is one beat: the first emphasized and the second released (compare the different intensity curve in Figures 4 and 5, between bars 171 and 175). This larger scale gesture is another factor that enabled the singer and the flutist to have the extra time they needed to accomplish this extemporized dialogue over the pianist's pedal. Indeed, this is the only moment in this section where the composer stops the movement of the baseline and the harmonic progression. One may speculate that the extemporized enhancement performed by the singer and the flutist, with the crucial support of the pianist, amplifies and makes more explicit the composer's intention at this point.^4^

Even if the members of the trio would have decided to try to, there wasn't enough time to plan the details of such a complex chain of events involving all three performers abandoning the conventional route of following the score's instructions. Listening to the recording after the performance, they were surprised to discover this moment in the improvised performance version, which suggests it was done without full awareness of the details.

No deviation from the score occurs in the prepared version, where in the same passage there is a mild increase of tempo during the first two beats of bar 172 (contrary to the decrease of tempo in the improvised performance).

The six described types of difference between the improvised and prepared versions of this Schubert movement, are closely similar to those found in Dolan et al. (2013) through analysis of performances of works by Telemann and Ravel, even though the compositional periods and languages (and the actual musicians undertaking the performance) were different. This lends support to the notion that the improvisatory state of mind enables a particular constellation of performance features which can be applied to music of varying styles; these features include the use of larger phrasing units, a greater range of dynamic and timbral changes, less emphatic metrical divisions, and extemporized gestures, spontaneously split and shared between partners, with the risk taking it represents (cf p. 32–33 of Dolan et al., 2013).

### Post-performance assessments

The performers were invited to reflect on their performances and the performance process a few days after the concert-experiment and again 20 weeks later. Rosie Bowker (flute) made a written account summarizing these responses, which can be read in full in Appendix 1.

The reflections followed critical listening sessions involving the three performers, facilitated by the first author. In the first reflections, the memory of the subjective experience was relatively fresh and present. The experience of watching and listening enabled the musicians to re-live the experience and retrieve some of the subjective experiences of the performance. Twenty weeks weeks later, the memory of the experience was more remote. Therefore, the critical listening was more focused on the musicians' considered assessment of the features present in the audio-visual recordings, as well as reflections on the nature of the contrasting mind-sets in the two types of performance.

The question discussed by the performers during the first series of the critical listening sessions was: “*How would you describe the differences you felt as performers, before and while performing, between the two mindsets?”*

In response, the musicians reported that the prepared version had to do with “…*greater feeling of mental and physical control… and being more precise about counting and note values… Overall the increased control resulted in a performance in which we played more consistently together within each bar because we were playing more in time, metronomically speaking*.” This corresponds with our findings about more emphasis in the prepared versions on shorter-term beats of quavers and crochets evenly emphasize (rather than whole bars or hyper-measures of two bars).

In the improvised version, where our analysis found larger beat and freer and longer-term phrasing, the musicians reported—“… *the freedom of the ‘let go’ mindset allowed me to create a wider range of colors and dynamics…*” This is confirmed by the analysis of the performances in section Sonic and Performance Related Parameters Characteristics of the Performances.

Twenty weeks later the author invited the musician to a second series of critical listening sessions, asking the following question: “*Please, could you share your thoughts about the performances and how you feel about them when you listen to the performances now, 20 weeks later?”*

In response, the musicians confirmed their perception of “*…a greater range and variety of timbre, dynamics and colors”*.

Further comments about the two modes of performance were in terms of performance attitude, artistic outcome, and well-being. One important feature was the sense of connection between the players. In the prepared/strict version performers got the experience of: “…*. listening to individual performers one at a time and reported having very little sense of connection between the performers*”. In the improvised version—“*When listening back to the ‘let go’ performance all of us responded to the video by saying that the performers were more integrated — there was a greater sense of connection and the ensemble work was more convincing*.”

The musicians noted the sense of trust that was manifest in the improvised performances, e.g., “*Trust in my own musical instincts and the capability to complete the task…”* They asserted that “*Trust between performers is imperative for being able to apply an improvisational state of mind …”*

A final feature related to experienced well-being/anxiety, e.g., “*If the trust isn't there between performers it becomes increasingly difficult to stay in the ‘let go’ mindset and much easier to revert to the ‘strict’, controlled and anxious mindset.”* Another related comment was “*the ‘strict’ mindset also resulted in Thibault and I reporting more self-conscious performances, increased levels of performance anxiety and more internal critical chatter”*. These statements are very consistent with the reported experiences in states of flow, and suggest that these states are conducive (possibly even necessary) to the kinds of performance characteristics observed.

### Continuous body motion tracking

#### Methods

We utilized Microsoft Kinect v2, a commercial motion tracking device, providing computer vision based motion sensing via mature APIs (Zhang, 2012). This version can provide data of up to 25 joints per body, with an improved tracking accuracy due to an enhanced depth sensor. By means of a scalable data fusion system, we could concurrently gather information from 5 Kinects sensors, improving the data resolution and overcoming some limitations such as occlusion when several bodies are together in the space.

We judge that wearables and wearing markers are generally more accurate than purely computer vision systems. However, the former are generally more cumbersome and might affect the performance. Although most research in this area has been focused on the fine-grained movements of fingers, wrists, or lips (Grosshauser et al., 2015; MacRitchie and McPherson, 2015), our research goals nevertheless focused on the broader head and body movements and in their comparison with performing styles. Putting that together with the need of a non-intrusive setup, makes the Kinect setup the most appropriate and cost-effective solution.

#### Data

The data collected through this system regarding motion consisted of a multivariate time series for each one of the detected bodies. Each multivariate time series is composed of 25 variables corresponding to the 3D positions of 25 joints that Kinects v2 can detect.

The recorded data was, unfortunately, heavily affected by noise due to imprecisions of the Kinect tracking mechanism. Also, due to the Kinect aligning system, the data points were sampled at irregular times, having no fixed sampling frequency. In order to reduce the impact of these impairments, the data was pre-processed as follows:

1. All data-points occurring within time bins of 250 ms were grouped together, and their median was taken as a representative. This generate time series with a regular sampling frequency of 4 Hz.
2. Positions were transformed into velocities, and all velocities above a given threshold were rejected as artifacts.
3. The joint velocities were grouped together (adding the magnitude of the 3-dimensional vectors) in 3 groups: head, upper body and lower body.

For the results described in the rest of this section, we have solely used the movements of the singer and the flutist. We chose them because they were the only two individuals with freedom to move their feet and move around, in contrast with the pianist and audience members who remained seated.

#### Statistics

The mean power spectrum and coherence between signals was computed using the well-known Welch method, using Fourier windows of 16 samples. The spectrum of velocities was divided in slow movements (below 0.75 Hz), medium movements (0.75–1.25 Hz) and fast movements (above 1.25 Hz). Also, linear regressions of the movements of one musician given the other's movements were computed over sections of 100 samples. Statistical significance is calculated using unpaired *t*-tests, and effect sizes are measured with Cohen's d.

#### Results

**Figure 6:**
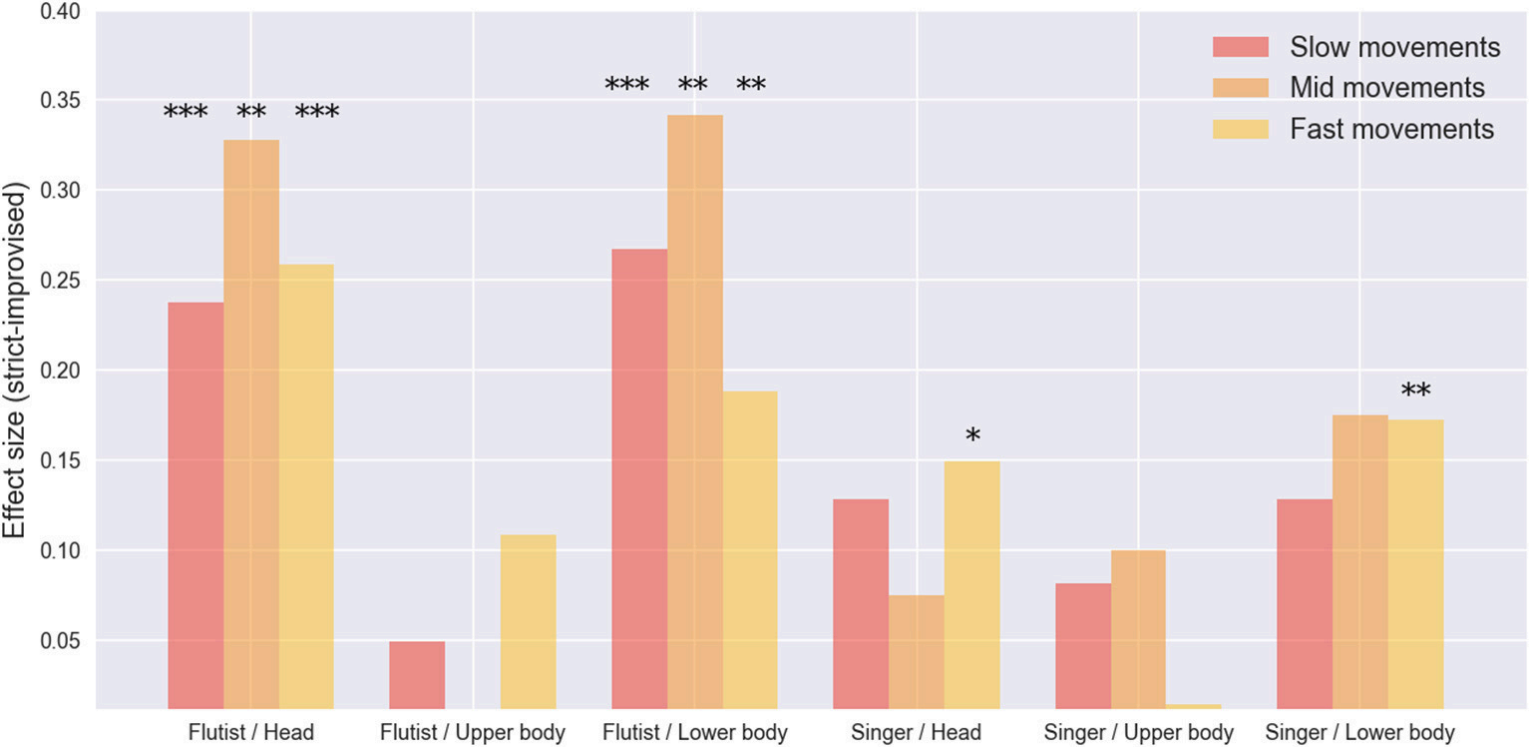
Effect size for movement differences between prepared (strict) and improvised (let-go) performances for the flautist and singer. *p < 0.05, **p < 0.01, ***p < 0.001.

We investigated the variations in the amount of movement in each musician between the prepared and improvised renditions, as given by the mean value of the total velocity of each of the three body segments. We found a consistent increase in movement in the prepared version, being significant for the fast movements of the head and lower body of the singer and all comparable movements of the flutist (see Figure 6).

**Figure 7:**
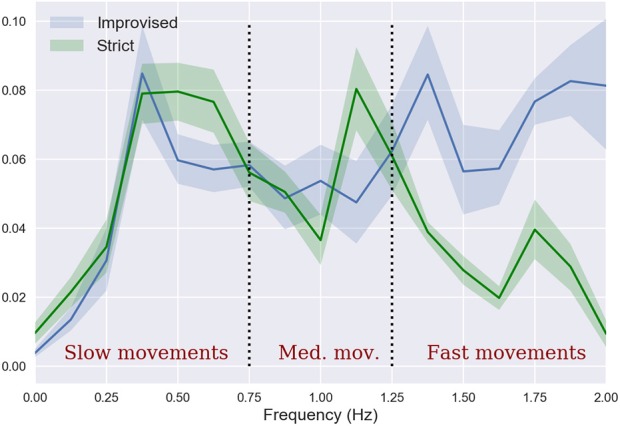
Covariance between movements of flutist and singer, decomposed by spectral components.

When studying the covariance and Pearson correlation coefficient between velocities of the body segments of flutist and singer we found no significant differences. However, when decomposing the covariance in its spectral components, we found that the correlation between the fast component of motion is markedly different during the improvised and prepared performance modes (see Figure 7). In particular, fast movements tend to be less correlated in the prepared (strict) than in the improvised (let-go) versions. Note that the coherence is a normalized quantity, and hence is not affected by changes in the total amounts of movement, making this finding independent of the previous one. Moreover, these two findings together imply that when shifting to the improvised performance the musicians' movements are reduced, and an important part of this reduction takes place over fast uncorrelated movements.

**Figure 8:**
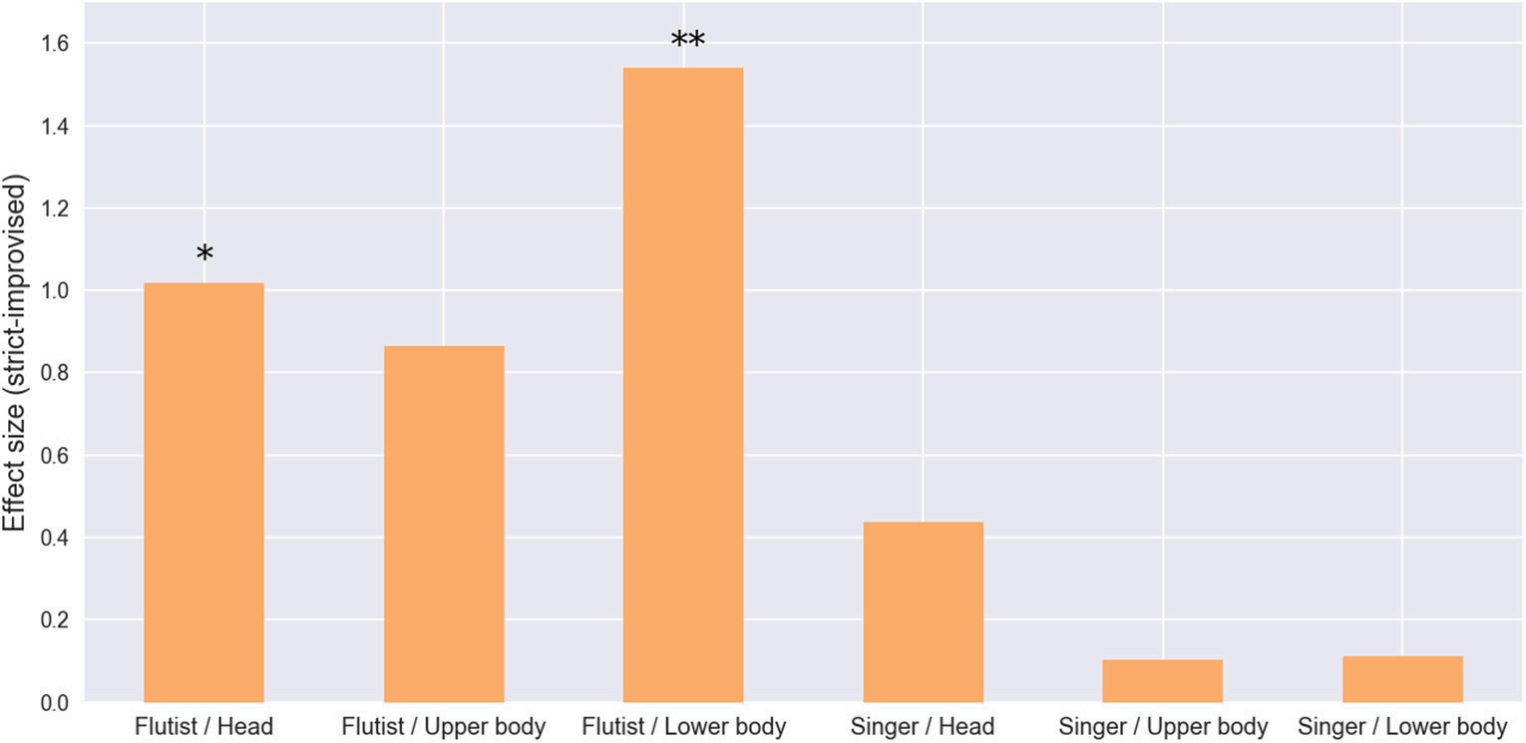
Residuals from linear regressions of flutist and singer given the movements of the other musician. *p < 0.05, **p < 0.01.

Finally, by comparing the residuals obtained after running a linear regression over the movements given the movements of the other musician, we found that on average all the residuals are larger in the prepared version, this difference is significant for the head and lower body movements of the flutist (see Figure 8). The consistency of this result supports our previous explanation, providing additional evidence toward the idea that an important cause of the additional movement found in the prepared version is due to movement that is not coordinated between musicians.

### Post-performance audience ratings

Levels of musical engagement/training were assessed through seven scaled items adapted from the Goldsmith's Musical Sophistication Index (Müllensiefen et al., 2014). These assessed, number of musical instruments played (including voice), amount of practice on these instruments, amount of formal training in music performance and music theory, and amount of listening to music (both recorded and live). A composite measure of engagement was obtained by adding these 7 scores together. These scores ranged from 6 to 33 with a mean of 22. Participants scoring 22 or less (*n* = 10) were assigned to the “lower engagement” group, those scoring 23 or more to the “higher engagement” group (*n* = 12).

Two-way ANOVAs were undertaken for each of the five post-performance ratings, with performance type (prepared or improvised) as a within-subjects factor, and level of musical engagement as a between-subjects factor. There was a significant main effect of performance for “emotionally compelling” [with the mean rating for the improvised performance being 3.8, as compared to the prepared performance at 2.6 (*F*_(1, 20)_ = 13.6, *p* < 0.001, Eta squared = 0.259)]. There was also a significant main effect of performance for “musically convincing” [with the mean rating for the improvised performance being 4.1, as compared to the prepared performance at 3.2 (*F*_(1, 20)_ = 7.4, *p* = 0.01, Eta squared = 0.320)]. There were no significant main effects or interactions involving the engagement variable, thus indicating that musical experience/training was not a significant influence on audience judgment.

Familiarity with the music of Franz Schubert was assessed by a single 4-point scale question, ranging from “not at all familiar/don't know” to “I know his music very well (i.e., possess recordings/have studied it).” 13 participants were assigned to the high-familiarity group (scoring 3 or 4), and 9 participants to the low-familiarity group (scoring 1 or 2).

Two way ANOVAs were undertaken for each of the five post-performance ratings, with performance type (prepared or improvised) as a within-subjects factor, and familiarity with Schubert as a between-subjects factor.

Table 1 shows the mean ratings in each condition. In addition to significant main effects of performance on “emotionally compelling” and “musically convincing” there was also a significant main effect of familiarity with Schubert on the “musically convincing” rating. Audience members who were familiar with Schubert rated the performances as less musically convincing (mean = 3.3) than those unfamiliar with Schubert [mean = 4.1, *F*_(1, 20)_ = 6.8, *p* < 0.02, Eta squared = 0.088]. There was also a significant interaction. For the dimensions of “emotionally compelling” [*F*_(1, 20)_ = 10.0, *p* < 0.005, Eta squared = 0.054] audience members familiar with Schubert showed a significantly greater difference in mean rating between the two versions (prepared = 2.6, improvised = 4.0) than those unfamiliar with Schubert (prepared = 4.0, improvised = 4.2).

**Table 1:** Mean audience ratings (max = 5) on five assessment scales, according to performance mode and familiarity with the music of Franz Schubert.

**Measure**	**Low familiarity with Schubert**		**High familiarity with Schubert**	
	**Prepared**	**Improvised**	**Prepared**	**Improvised**
Improvisatory	2.0	2.5	1.4	2.1
Innovative	2.2	2.9	1.5	2.1
Emotional	3.4	3.7	1.9	4.0
Musical	4.0	4.2	2.6	4.0
Risk-taking	1.8	2.6	1.6	2.3

Finally, two way ANOVAs were undertaken for each of the five post-performance ratings, with performance type (prepared or improvised) as a within-subjects factor, and with the presence or absence of sight of the performers as a between-subjects factor. In no case was there a significant effect of sight, either as a main effect or in interaction.

In sum, two of the post-performance rating scales (“emotionally compelling” and “Musically convincing”) were sensitive to the differences between the prepared and improvised version, with the improvised version rated higher than the prepared version. This effect did not depend on whether the audience members could see the performers, nor was it affected by the level of musical training of audience members. Familiarity with the music of the composer did, however, impact on the results. Those familiar with Schubert judged the improvised version more emotionally compelling when compared to the prepared version, than did those unfamiliar with Schubert.

### Real-time continuous monitoring of brain activity (EEG) of performers and audience members

#### Methods

##### Data acquisition

Raw EEG signals of the three performers and four audience members were measured using CE-certified devices (NCLogics AG, Munich, Germany). For each participant, 19 Ag/AgCl electrodes were placed on the following locations (all according to the 10–20 electrode position system; Klem et al., 1999): Fp1, Fp2, F3, F4, F7, F8, C3, C4, T7, T8, P3, P4, P7, P8, O1, O2, Fz, Cz, Pz. The reference electrode was placed behind Cz and the ground electrode on the forehead. All locations were cleaned with abrasive gel and conductive gel was used to ensure low skin impedance. EEG data were collected at 250 Hz, and bandpass filtered between 2 and 40 Hz. All devices were synchronized via a local Wifi network. Start and ending of each measurement were remotely controlled and synchronized. Times series EEG data were stored and exported for further analysis. Bad channels and bad epochs were visually identified and removed from the analysis.

##### Signal complexity

The method for calculating the LZ consists of two steps. First, the amplitude of a given signal X of length T is digitalized, calculating its median value and turning each data point that is above it to “1”s and each point below it to “0”s. Then, the resulting binary sequence is scanned sequentially, looking for distinctive structures that are used to form a “dictionary of patterns.” Finally, the signal complexity is determined by the number of patterns that compose the dictionary, denoted by c(X). Note that regular signals can be characterized by a small number of patterns and hence have low LZ complexity, while irregular signals with no characteristic patterns requires long dictionaries and hence have large LZ complexity. Moreover, the quantity

$$\begin{array}{l} \frac{\text{c}\left(\text{X}\right)\text{log}\left(\text{T}\right)}{\text{T}} \end{array}$$

is an efficient estimator of the entropy rate of X (Ziv, 1978), which has various interpretations within information theory (Cover and Thomas, 2012) and thermodynamics (Mézard and Montanari, 2009). This makes this normalized LZ a principled, data-efficient and timescale-independent estimator of the diversity of the underlying neural process. In the rest of the manuscript we refer to the quantity in the formula above generically as LZ.

##### Statistics

The neural signal was split in segments of 2 s, which provides enough data points to have an accurate estimation of LZ while being short enough to keep safe the stationarity of the data. The values of each segment were then binarized using the corresponding median value as a threshold. The LZ was finally calculated for each temporal segment of each electrode, and then averaged across time and electrodes to obtain one LZ value per subject per condition. Due to our small sample sizes, statistical significance is determined with *t*-tests (paired when possible, and unpaired elsewhere) and effect sizes are measured with Cohen's d.

#### Results

##### Increased complexity in the improvised version

**Figure 9:**
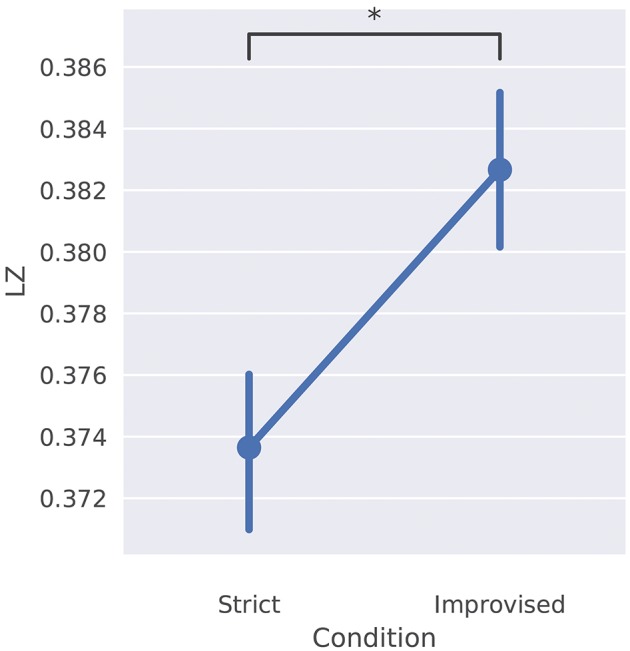
Overall LZ for each condition, averaged across participants and channels. *p < 0.05.

**Figure 10:**
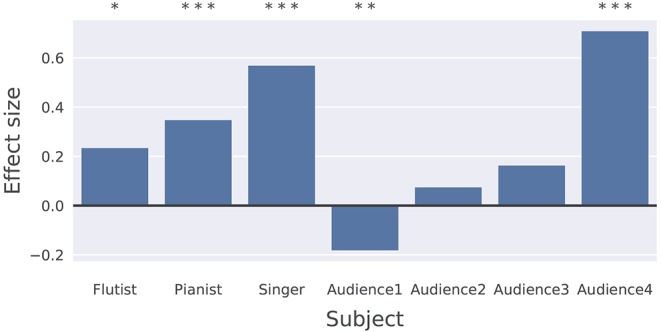
LZ effect size (calculated using Cohen's *d* for each individual. *p < 0.05, **p < 0.01, ***p < 0.001.

Based on the properties of LZ outlined above, we investigated the complexity of the measured EEG signals of the three performers and four audience members in both conditions, under a working hypothesis that LZ is higher during the improvised than during the prepared condition. Our main result is that LZ increases in the improvised condition with respect to the prepared condition by a difference of 0.009 (95% CI: 0.001–0.016, *n* = 7, *p* = 0.031), shown in Figure 9. Significance was calculated using a two-sample (i.e., paired) *t*-test. Figure 10 contains the effect sizes (Cohen's d) for each participant with subject-level significance calculated using a Mann-Whitney *U*-test.

The small *p*-value for the group-level test is caused by the fact that the observed LZ increase is very consistent across subjects, with 6 of the 7 participants showing changes in the same (positive) direction. While results among the audience are mixed, all three musicians show substantial increases in LZ during the improvised performance, and this effect is most significant in the singer and the pianist (see Figure 10).

##### Complexity increase comes from the right brain hemisphere

**Figure 11:**
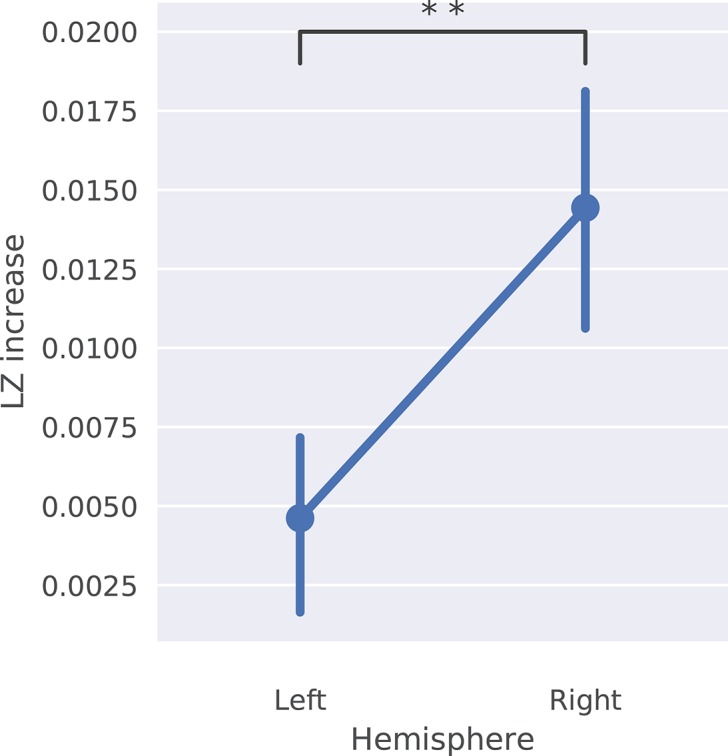
Average LZ increase between conditions observed in all channels in the left and right brain hemisphere. **p < 0.01.

**Figure 12:**
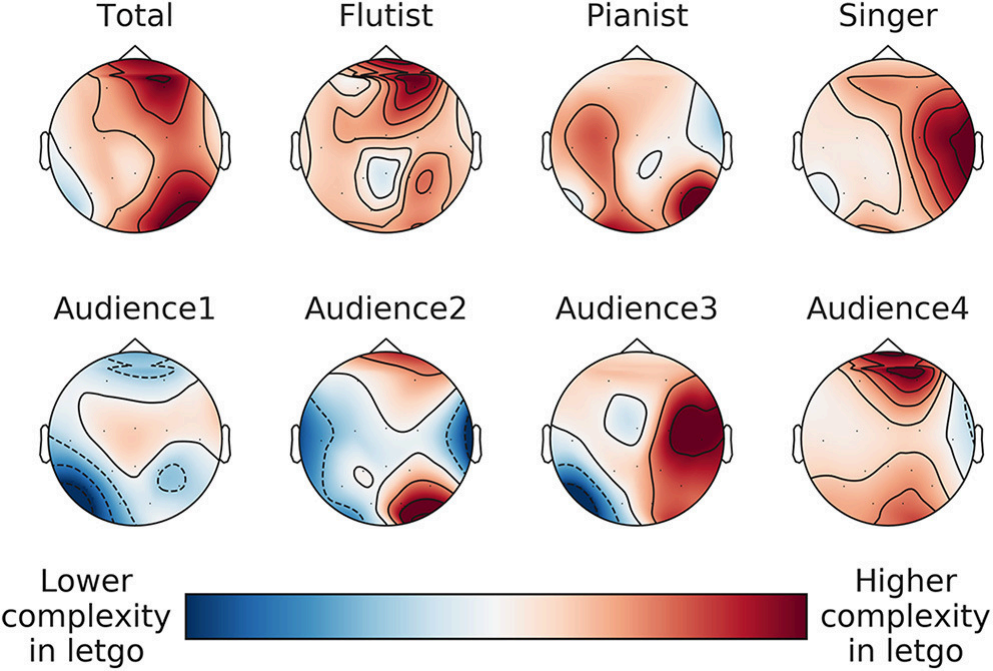
Topological map of the LZ increase between the “strict” and “improvised” renditions.

Following up on our main result, and in agreement with accepted neuroscientific theories, we find that the LZ increase is mainly localized in the right hemisphere (average difference in LZ increase between right and left hemisphere: 0.01, 95% CI: 0.004–0.016, *p* = 0.003). The right hemisphere is conventionally associated with cognitive processes like creativity and divergent thinking, which indicates that musicians were more engaged in a creative process during the improvised performance, and were less likely to enter the logic-driven and rule-following states usually associated with the left hemisphere. Figure 11 shows the average difference in LZ increase and Figure 12 its spatial distribution.

##### Changes in EEG power spectrum

**Figure 13:**
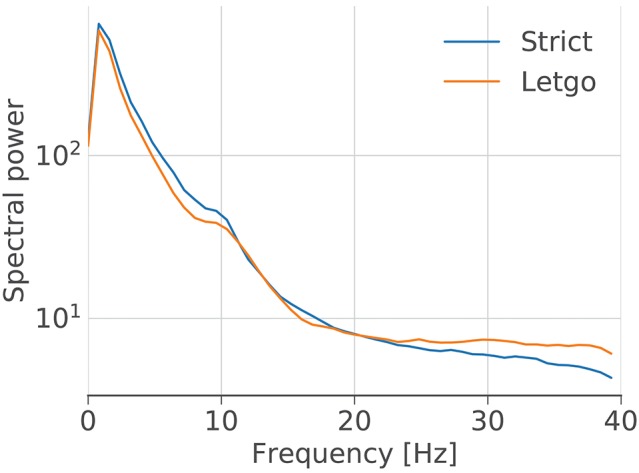
Aggregated power spectrum. Power spectrum averaged across all subjects, calculated using Welch's method.

We also calculated the average power located in each frequency band of the EEG signals of musicians and audience in the two conditions. We found that during the prepared performance there is more power located in low frequencies (delta, theta and alpha bands), while high frequencies (beta and gamma bands) are more active during the improvised mode. Interestingly, a similar phenomenon has been found when comparing EEG data from sleep conditions: high frequencies exhibit relatively more power during REM sleep and low frequencies are relatively more active during unconscious, dreamless sleep (Achermann et al., 2016). This suggests a relationship between this “crossed spectrum” (as shown in Figure 13) and various degrees of awareness, providing additional evidence to support the hypothesis that musicians and audience are more aware during the improvised performance than in the prepared version.

### Discussion

This study confirmed distinct differences between prepared and improvised approaches to performances of the same piece of music. These differences were revealed through complementary analyses of (a) objective characteristics of the sound recordings, (b) musicians' self-report, (c) musicians' movements during the performances, (d) listener ratings, and (e) EEG measurements on both performers and listeners.

We take each of the detailed research questions in turn and briefly discuss what light our research has shed on each of them.

**Do Performers' Subjective Accounts of Their Improvisatory Experiences Contain Elements Indicative of a Flow Experience?**

The fact that the musicians reported surprise at discovering what they had done, suggest that to some degree, their actions were driven by intuition, and accessing knowledge in a non-conscious-analytical way, rather than conscious planned decision. Moreover, during the improvised rendition the performers took a significant number of risky choices and yet the results sound more coherent, while the musicians experienced less anxiety and effort, and more pleasure.

**Are There Quantitative Signatures of a Shift From a Secondary Toward a Primary State of Cognition When Comparing the Brain Activity During the Prepared and Improvised Performances? In Particular, can one Find Significant Differences in Terms of the LZ Complexity of the EEG Signals of Musicians and Audience?**

While the literature about states of flow is mainly based in psychology, discussion of the EBH are mainly rooted in neuroscience. We link these two previously disconnected literatures by raising the tentative idea that *all states of flow are primary states* (but not vice-versa). In other words, all the descriptions associated with feelings of flow are consistent with the characteristics of primary states of cognition, while it is clear that not all primary states involve flow.

Currently, mainly because of their epistemological origins, the presence of states of flow and primary states are generally established by different but complementary methods: primary states are related to high entropy in brain functions to be found in quantitative properties of neural measurements, while states of flow are found by subjective reports. Some effort toward finding biomarkers of states of flow have been reported in a study undertaken with theatre artists (Noy et al., 2015). That study presented kinematic (CC motion) and physiological evidence (heart rate and subjective ratings) consistent with the subjective reports of the artists. Our study reveals that LZ complexity is one such potential marker. However, further experimental evidence will be required to fully corroborate this claim.

In this multidisciplinary study, using standard methods from computational neuroscience and psychology we provided evidence that the improvisatory state of mind in musicians can be conceived of as both a primary state and a state of flow, as would need to be the case if all states of flow are primary states.

The identification of the improvisatory state as being a primary state is supported by the higher level of LZ complexity found in the EEG signals recorded during the improvised performance. Moreover, the LZ increase was mainly localized in the right hemisphere, suggesting more engagement in a creative process during the improvised performance. The LZ effects were further supported by the profile of the power spectrum found in the EEG signals of the prepared and improvised performances, which resemble the transition between sleep and awake states as reported in the literature.

Characterizing the improvisatory state of mind as involving elements of a state of flow is supported by the musical analysis, which reveals features of the improvised performance such as longer-term phrasing gestures, and “mind-reading” in the passing of improvised gestures from one to the other. This is supported by the audience ratings, which found the improvised performance more emotionally compelling and musically convincing. An additional element that supports the state of flow in the improvised performance is the existence of longer and more flowing musical gestures, which are suggested by both the musical analysis and the reduced amount of uncorrelated fast movements in the motion analysis. The features of the performance found in these performances of Schubert are similar in nature to the features discovered in an earlier study when different musicians performed music by Telemann and Ravel, thus suggesting that these are quite general, high level, features of the improvisatory approach (beyond particular stylistic devices of different historical periods, or specific performers).

A significant question about the improvisatory state of mind, not previously addressed in the literature, is whether it is transferable from musicians to audience members. The results obtained from the EEG measurements and the psychological questionnaires both suggest this transfer is possible, although the fact that only three out of four audience members showed the LZ effect demonstrates that other factors not measured here (e.g., focus of attention) may intervene. Interestingly, our results suggest that this transfer is not affected by visual aspects of the performance, as the most heavily affected audience member was actually blindfolded and hence only listening to the performance. Moreover, the fact that there was less movement displayed by the musicians during the improvised than during the prepared version, and the fact that the correlated movement do not increase significantly, suggests that the causes of the change in brain activity of the audience is not due to the musicians' movements. Moreover, musical training seems not to affect the transfer of the improvised state of mind to an audience, since the effects shown both by questionnaire and also by EEG measurement were present in people with both higher and lower levels of musical training. This is encouraging, as it suggests that this experience is open to a broad range of people, not just those schooled in formal elements of musical language. This may suggest that the phenomenon is driven in part by underlying universal elements of expression (Cohen and Inbar, 2002; Godoy and Jorgensen, 2012). Further support for the relevance of reference to universal elements of expression is the re-appearance of similar gestures of musical expression by different musicians, performing different musical styles to different audiences in two different studies, when the performance consisted of improvised approach. It is therefore tempting to say that the improvisatory state of mind is a specific state of flow, which is in turn a specific kind of primary state. This would require however to find a specific difference that distinguishes the improvised state of mind from other states of flow. Three ingredients seem to be particularly distinctive of group musical improvisation: real-time creativity, shared risk-taking, and a feeling of enhanced listening/togetherness. This latter phenomenon has been explored in the context of movement interaction (Noy et al., 2015), and also in collective musical performance (Müller and Lindenberger, 2011). Some recent studies have also reported inter-brain synchronization between musicians that are performing togsether (Sänger et al., 2012, 2013; Müller et al., 2013). The statistical framework used in Dumas et al. (2010) is appropriate for such explorations. However, the experimental protocol of the current study was not suitable for exploring this issue in the current data. The approach taken here does however offer the prospect of discovering further commonalities across improvising performers, and between performers and audience.

**Do the Body Movements of Musicians as Visually Experienced by Audience Members Affect the Magnitude of Their Response to the Improvised Performances?**

Musicians moved significantly less during the improvised performance in comparison to the prepared performance. Since both EEG complexity and audience ratings increased for the improvised performance, these increases could not be attributed to more body movement. This is confirmed by the comparison between those audience members both seeing and hearing the performance, and those only hearing it. Seeing the performers made no significant difference to the response. A plausible explanation for the lower level of movement in the improvised performances is that such movements are linked to prominent metrical beats. The analysis of the performances' sonic characteristics has shown that the improvised performances emphasize longer beats (“hyper-measures”) and de-emphasize individual, shorter-term beats.

**Does the Level of Musical Training or Knowledge of Audience Members Affect Their Response to the Improvised Performances?**

Some post performance rating scales were sensitive to the differences between the prepared and improvised version, with the improvised version rated higher than the prepared version. This effect did not depend on the level of musical training of audience members. However, those familiar with Schubert judged the improvised version to be more emotionally compelling than did those unfamiliar with Schubert. Arguably this may be a response to “novelty,” as evidence exists that musical emotionality is linked to the level of unexpectedness of what is experienced (e.g., Steinbeis et al., 2006). For those unfamiliar with Schubert, both performances would be relatively novel. For those familiar with Schubert, the improvised version would be experienced as more novel than the prepared version.

**Do the Objective Performance Characteristics That Distinguish Improvised Performances of Telemann and Ravel Extend to the Music of a Different Period Exemplified by Schubert?**

The analysis of improvised performance characteristics shows a significant convergence across three separate classical periods, in a common more free use of timbral variations, and longer temporal and dynamic units, which de-emphasize individual beats and bars, as well as showing more “mind reading” and risk-taking between performers. This gives us some confidence that we are tapping quite general features of the improvisatory approach which at least to some extent transcend genres and periods, and may therefore reflect more universal features of human behavior, consistent with the postulated existence of a biologically universal primary state which is to some extent driving behavior during the application of an improvisatory approach.

In addition, there is a strong suggestion both from the audience responses (of “more musically convincing”) and also from the critical listening of the musicians, that the improvised performances were not only more impactful, but had a higher artistic quality.

## Concluding remarks

The research we have presented indicates that improvisation is related to a special state of mind, both amongst the performers and their listeners. The creation of music and its appreciation is a highly multifaceted phenomenon, and therefore developing insight about its nature necessitates research that combines assessments of physiological, psychological and interpersonal communication. We believe that an improved integrated understanding of psychological and neuroscientific aspects of improvisation is of fundamental importance.

The current increase in the number of mental health cases that our society is experiencing may be related to a lack of ability to apply an improvisatory attitude during a daily life that becomes ever more unpredictable. To study how classical musicians are able, at will, to switch between improvised and non-improvised performance modes presents a unique opportunity, in which a careful comparison between these two ways of behaving can be carried out. What we noticed may suggest that, unlike the prepared performances, in improvisatory state of mind the musicians aim spontaneously toward the macro-structure, while the “local” tasks are performed more successfully, with less effort and anxiety, and in full accordance with the definition of a flow state presented in Csikszentmihalyi (1975, 1997).

It would be interesting in future research to develop measurement techniques that are minimally intrusive though still allow recording of both individual and collective brain, body and psychological responses during concerts. The closer the research can get to a real-life concert situation, the more relevant the findings become, as the corresponding objective and subjective findings might better reflect fundamental elements of human experience. These insights might contribute to deepen our understanding of the musical experience, which in turn can help to improve artistic and pedagogical praxis. Moreover, we hope that our findings can motivate further investigations on the effects of improvisation in well-being, potentially relevant to the links between performing arts and therapy.

## Ethics statement

The experiments reported in our manuscript were part of the protocol approved by the Ethics Committee of Guildhall School of Music and Drama. A separate ethics approval for the research reported in our manuscript was not required as per Imperial College Research Ethics Committee's guidelines as well as national regulations.

## Author contributions

DD designed the concert experiment (the performance followed DD's approach to classical improvisation and its applications on performance), analyzed the performances and contributed to the writing process. HJ took the overall lead on the design and analysis of the neuroscience component of the study, and contributed to writing process. PM-M contributed to the pre-processing and analysis of EEG data. MM-S contributed to the setup of the experiment and its logistics; gathered, managed and processed the movement data. HR contributed to the analysis of EEG data. FR contributed to the analysis of the EEG data and the movement data, to the interdisciplinary analysis, and to the overall writing process. JS designed and analyzed the audience questionnaire, contributed to overall project design and management, and led the drafting process.

### Conflict of interest statement

The authors declare that the research was conducted in the absence of any commercial or financial relationships that could be construed as a potential conflict of interest.

## Footnotes

^1^We thank Robert Levin for referring us to this document.

^2^The Sonic Visualizer was developed in Queen Mary University of London as a part of the CHARM project. We thank the double-bass player and researcher, Mark Gilenson (Schola Cantorum, Basel), for his assistance with the Sonic Visualizer analysis.

^3^The term Hyper-measure, attributed to Cone (1968), refers to groups of bars, where bars act as beats, leading to a larger-scale basic rhythmic gesture. A generation before Edward Cone, the highly influential pianist and teacher Arthur Schnabel, used this concept and terminology in his teaching. (Cone studied with Arthur Schnabel's son, Ulrike Schnabel).

^4^The score attached is the performers' working draft. What is marked here as bar 1 is in fact an upbeat, meaning that bar 1 is actually the bar marked as 2 in the musicians' working score. Since the musicians worked with this score, we will refer to their markings. The Sonic Visualizer graphs refer to the musicians' working score bar numbers. In this performance the part written for clarinet in B was played by a flute in C. This means that all flute pitches referred to in the text are one whole tone lower than what is notated. and the performance began at bar 7.

## Supplementary material

Video 1 Bars 7–8 (flute solo opening 3 notes) prepared (strict) version.

Video 2 Bars 7–8 (flute solo opening 3 notes) improvised state of mind version.

Video 3 Bars 8–12 prepared (strict) version.

Video 4 Bars 8–12 improvised state of mind.

Video 5 Bars 165–177 prepared (strict) version.

Video 6 Bars 165–177 improvised state of mind.
