# Supplementary material for: The Improvisational State of Mind: A Multidisciplinary Study of an Improvisatory Approach to Classical Music Repertoire Performance
Source: Front Psychol. 2018 Sep 25;9:1341. doi: 10.3389/fpsyg.2018.01341 (PMC6167963; doi:10.3389/fpsyg.2018.01341)
Supplement: Supplementary file 7 [file Data_Sheet_1.docx]

**Appendix 1**

What follows is an exact transcript of the written commentary of flautist Rosie Bowker, summarising and commenting on the two feedback sessions involving all performers and the first author.

**Part 1: Performers’ immediate responses:** *Question from author: “How would you describe the differences you felt as performers, before and while performing, between the two mindsets - ‘strict’ versus ‘let go’ or ‘improvisational state of mind’”?*

In our written feedback to this question there was a consensus between the three of us. In the ‘strict’ versions we recounted a greater feeling of mental and physical control including trying to control technical aspects of our playing and being more precise about counting and note values. Each of us focused on our individual parts, anticipating and planning the next section of the music. Overall the increased control resulted in a performance in which we played more consistently together within each bar because we were playing more in time, metronomically speaking. However, the ‘strict’ mindset also resulted in Thibault and I reporting more self-conscious performances, increased levels of performance anxiety and more internal critical chatter.

In the ‘let go’ mindset we all reported a stronger sense of connection between the three of us. We looked at each other more and looked at the music less. We listened better to each other and responded to each other in the moment. Kate wrote that she “found [herself] attending more to the meaning of the words, and letting [her] imagination respond more to what [she] heard… [She] could begin to communicate the sense of being in a different place, embodying different emotions, and responding more in the moment to changes in the music, listening and reacting more.” For me the freedom of the ‘let go’ mindset allowed me to create a wider range of colours and dynamics. For Thibault it allowed him to feel more freedom and flexibility in the tempo and performance.

**The two mindsets**

The most important thing to say about the two mindsets is that the ‘strict’ mindset is not synonymous with performing badly or unmusically. The ‘strict’ mindset can also be thought of as the ‘trying to win’ mindset with the emphasis on the word ‘trying’. This mindset comes when a performer really wants to perform her/his best. The performer puts herself/himself under internal and external pressure and tries hard to control every sound produced and every technical aspect of playing.

Likewise, the ‘let go’ mindset is not synonymous with playing better or more musically. The ‘let go’ mindset allows the performer to take risks and make mistakes without worrying about internal or external pressure. The performer uses the score as a starting point for interpretation of the music. As a performer I often find that the ‘let go’ mindset comes naturally in performances where I have nothing to lose. In this mindset I can step into the room with the aims of enjoying myself and having fun with the music. This can be done to a greater or lesser degree – in an audition a performer can use the ‘let go’ mindset or improvisational state of mind without changing a single note.

**A link between the two mindsets?**

“In Thibault’s written response immediately after our performance-experiment, he wrote that “I am convinced this close attention for detail allowed our trio to be the most free it has ever been in the second rendition of the Schubert.” One possible conclusion to draw from this statement is that the ‘strict’ mindset can also be seen as a ‘preparatory’ state of mind or an initial step that performers can use to ensure that they can rely on and trust each other when they use the ‘let go’ mindset in performance.

**Part 2: Responses when listening to and watching the audio and video recordings 20 weeks later.**

*“Question from author: “Please, could you share your thoughts about the performances and how you feel about them when you listen to the performances now, 20 weeks later?”*

**“**There was a strong consensus between Kate Smith, Thibault Charrin and I when listening back to the ‘strict’ performance. We each wrote about listening to individual performers one at a time and reported having very little sense of connection between the performers. The ‘strict’ performances were very precise and together (I reported counting along to the music). Kate wrote that she “found [herself] listening to the minutiae of the music… [She] was drawn to accuracy rather than [to the] story.”

“When listening back to the ‘let go’ performance all of us responded to the video by saying that the performers were more integrated – there was a greater sense of connection and the ensemble work was more convincing. We all reported a greater range and variety of timbre, dynamics and colours. Kate and I wrote that as listeners we were more engaged: the ‘let go’ version was more exciting because of the unexpected moments including some where the performers were not together. The longer musical shapes allowed us as listeners to relax and we were able to see that we - the performers - were having fun!

“All of these responses mirrored what we reported immediately after the performance.

**Influence of the experiment on subsequent performances**

On further reflection since the experiment, all of the freedom that the ‘let go’ mindset allowed me individually was the result of one thing: a deep sense of trust. Trust in my own musical instincts and the capability to complete the task, trust in my knowledge of the piece that I was about to perform (preparation), and trust in the performers with whom I was sharing the stage.

Trust *between* performers is imperative for being able to apply an improvisational state of mind (the ‘let go’ mindset). It allows the performer to be brave and to know that the others will be there to catch her/him when (s)he tries something new in the moment. If the trust isn’t there between performers it becomes increasingly difficult to stay in the ‘let go’ mindset and much easier to revert to the ‘strict’, controlled and anxious mindset. The ‘let go’ mindset gives the performer a freedom to trust herself/himself and her/his fellow performers. It allows the performer to escape the internal and external pressures associated with performance.’
